# An imbalance between apoptosis and proliferation contributes to follicular persistence in polycystic ovaries in rats

**DOI:** 10.1186/1477-7827-7-68

**Published:** 2009-07-01

**Authors:** Natalia R Salvetti, Carolina G Panzani, Eduardo J Gimeno, Leandro G Neme, Natalia S Alfaro, Hugo H Ortega

**Affiliations:** 1 Departamento de Ciencias Morfológicas, Facultad de Ciencias Veterinarias, Universidad Nacional del Litoral, Argentina; 2 Centro de Experimentaciones Biológicas y Bioterio, Facultad de Ciencias Veterinarias, Universidad Nacional del Litoral, Argentina; 3 Instituto de Patología, Facultad de Ciencias Veterinarias, Universidad Nacional de la Plata, Argentina

## Abstract

**Background:**

Cystic ovarian disease is an important cause of infertility that affects bovine, ovine, caprine and porcine species and even human beings. Alterations in the ovarian micro-environment of females with follicular cysts could alter the normal processes of proliferation and programmed cell death in ovarian cells. Thus, our objective was to evaluate apoptosis and proliferation in ovarian cystic follicles in rats in order to investigate the cause of cystic follicle formation and persistence.

**Methods:**

We compared the number of in situ apoptotic cells by TUNEL assay, expression of active caspase-3 and members of Bcl-2 family by immunohistochemistry; and cell proliferation by the expression of the proliferation markers: PCNA and Ki-67.

**Results:**

The proliferation index was low in granulosa of tertiary and cystic follicles of light exposed rats when compared with tertiary follicles of control animals, while in theca interna only cystic follicles presented low proliferation index when compared with tertiary follicles (p < 0.05). The granulosa of cysts exhibited a similar cell DNA fragmentation to early atretic follicles. In the granulosa and theca interna, active caspase-3 shown similar immunostaining levels in tertiary and cystic follicles (p < 0.05). The granulosa cells presented high expression of Bcl-2, Bcl-xL and Bcl-w in the tertiary and cystic follicles with diminishing intensity in the atretic follicles, except with Bcl-w where the intensity was maintained in the atretic follicles (p < 0.05). The expression of Bax was weak in the healthy and cystic follicles. In the theca interna, Bcl-2 expression was the same as the pattern found in the granulosa; no differences were found between tertiary and cystic follicles from both groups for Bcl-xL and Bcl-w. The expression of Bax in this layer was higher in the tertiary follicles of the treated animals (p < 0.05) while the values for cystic follicles were similar to those in the tertiary follicles of controls. The theca externa showed low expression of the pro and anti-apoptotic proteins.

**Conclusion:**

These results show that the combination of weak proliferation indices and low apoptosis observed in follicular cysts, could explain the cause of the slow growth of cystic follicles and the maintenance of a static condition without degeneration, which leads to their persistence. These alterations may be due to structural and functional modifications that take place in these cells and could be related to hormonal changes in animals with this condition.

## Background

Folliculogenesis, ovulation, and the subsequent formation of the corpus luteum are intricate processes that involve dramatic changes in ovarian cell function. Once initiated, follicular growth is a continuous process without resting phases, ending at ovulation [1]. One of the main modifications in granulosa cell function is the rapid switch from the highly proliferative stage characterizing granulosa cells of preovulatory follicles to the non-proliferative, terminally differentiated phase of luteal cells. Cell cycle regulation is a complex process involving a balance between several regulatory molecules, and can be altered by numerous external signals in multiple steps [1]. Proliferating cell nuclear antigen (PCNA), and Ki-67 are proliferation-associated proteins. PCNA is required for DNA synthesis and appears to be involved in follicular growth. Expression of PCNA in granulosa cells begins upon the formation of a primary follicle, and its level of expression appears to increase during the gonadotropin-dependent stages of preovulatory follicular development [2,3]. Ki-67 is expressed in G1/S/G2/M but not G0 cells [2]. Both proteins can be detected by immunohistochemistry and they are excellent markers of cellular proliferation [1].

A fine balance between survival and apoptotic factors may determine whether a follicle will continue developing or undergo atresia [1,4-6]. The progression of apoptosis in follicular cells is dependent on the cooperative regulation of different paracrine and autocrine factors; it is likely that none of these factors is specifically required in the control of follicle growth or death. Regulation of apoptotic signaling in the ovary is generally achieved by Fas system and the Bcl-2 family [5,7,8]. Members of the Bcl-2 family of proteins are considered among the main regulatory proteins acting at the mitochondrial level. They can be divided into those having either an antiapoptotic (e.g. Bcl-2, Bcl-W, Bcl-x_L_) or proapoptotic (Bax, Bad, Bim, Bcl-x_S_, Bod, Bok/Mtd) function. The antiapoptotic proteins can block the activation of effector caspases, caspase-3, caspase-6 and caspase-7, which in turn transduce the apoptotic signals [9-11]. Bcl-2 resides on the nuclear/endoplasmatic reticulum membrane, with a smaller portion on the mitochondrial membrane, and, it has been suggested that when it is present in the outer mitochondrial membrane it can block apoptosis by inhibiting the release of apoptosis-inducing factors, cytochrome c and the intermembrane protein DIABLO-Smac from the mitochondria [11,12]. Bcl-xL and Bcl-w are usually abundant in the mitochondrial membrane where they inhibit the release of apoptotic inducing factors [13].

On the other hand, Bax plays a major role in initiating the release of cytochrome c. This protein may form a pore in the outer membrane of the mitochondria allowing cytochrome c to leak out. Therefore, the Bax: Bcl-2 (or Bcl-xL or Bcl-w) ratio might be important in the mitochondria-dependent apoptosis cascade for the release of cytochrome c and DIABLO-Smac [11,12].

Cystic ovarian disease (COD) and/or polycystic ovarian syndrome (PCOS) are disorders of the reproduction that affect bovine, ovine, caprine and porcine species and even human beings [14-18]. The heterogeneity of the syndrome is reflected in many animal models of polycystic ovaries (PCO) [17,19-22]. In the bovine livestock, COD is an important cause of infertility and it is characterized by anovulation, anoestrus, and the persistence of follicles with a larger diameter than the ovulatory follicle [14].

Several experimental models for PCO have been developed in rats [17,19-22]. Estradiol valerate (EV) has been employed to induce this syndrome and cause a sudden appearance of polycystic ovaries due to disturbances in metabolic and physiologic processes [19,20]. A simple method to induce the disease is to expose mature rats to an environment with constant light during 90 days. After this time, the females develop persistent follicles that remain while the environmental conditions persist [17]. Constant illumination acts on the hypothalamus to induce a fail in the preovulatory LH surge and ovulation is thus prevented, leading to the follicular persistence [17]. Such method induce cysts gradually, similar to the PCOS and is also the least invasive of all the methods developed till now.

Although it is widely accepted that dysfunction of the hypothalamic-pituitary-gonadal axis is an important aetiological factor of cystic follicles, delay of follicle regression after ovulation failure is an alternative cause of cysts [23]. Alterations that occur in the ovarian micro-environment of females that present follicular cysts could alter the normal processes of proliferation and programmed cell death in ovarian cells. Based on this, our objective was to evaluate apoptosis and proliferation in ovarian follicles in anovulatory COD rats compared with rats with regular oestral cycles in order to investigate the mechanisms underlying follicular survival or atresia. We compared the number of apoptotic cells by Terminal Deoxynucleotidyl Transferase Biotin-dUTP Nick End Labelling assay (TUNEL), activated caspase-3 and Bcl-2 family members expression by immunohistochemistry and cell proliferation by expression of the proliferation markers: PCNA and Ki-67.

## Methods

### Animals and treatment

All procedures were carried out according to the Guide for the Care and Use of Laboratory Animals [24]. Female Wistar rats (16 weeks-old), were provided by the Centre for Experimental Biology and Laboratory Animal Sciences (Facultad de Ciencias Veterinarias, Universidad Nacional del Litoral). Before the experiment, the animals were kept under a controlled cycle of light-darkness (lights on from 8:00 h to 20:00 h), at 18–22°C with free access to water and commercial food (Cargill, Argentina). Lighting was provided by banks of General Electric 4 coolwhite 40 W fluorescent tubes to obtain an intensity of 350 l× to 1 m from the floor.

Fifteen animals displaying at least two normal 5 day estrous cycles just prior to treatments were divided into two groups. A control group (*n *= 5) of females of the same age as the treated animals remained in the normal light-dark conditions; the continuous light group (*n *= 10) were placed in the conditions described except that the light cycle was extended to 24 h [17].

Smears obtained by vaginal washing were examined under a microscope for the relative abundance of nucleated epithelial cells, cornified cells and leukocytes [25]. Cycles with duration of 5 days were considered regular. The presence of cornified cells in the smears for a minimum of 10 consecutive days was defined as persistent vaginal cornification and considered as confirmation of follicular cystic development [17].

### Tissue sampling

The animals in the light exposed group were sacrificed approximately 2 weeks after persistent vaginal cornification was established; those in the control group were sacrificed after 12 weeks in proestrus to obtain healthy growing follicles.

After being anaesthetized with a cocktail of ketamine/xylazine (40/4 mg/kg) via subcutaneous injection, the rats were killed by decapitation; trunk blood was collected; and serum was stored at -20°C until being used for hormone assays. The ovaries were dissected and fixed in 10% (v/v) buffered formalin for 6 h at 8°C and were washed in phosphate buffered saline (PBS). For light microscopy, fixed tissues were dehydrated in an ascending series of ethanol, cleared in xylene, and embedded in paraffin. Five micrometer-thick sections were mounted in slides previously treated with 3-aminopropyltriethoxysilane (Sigma-Aldrich, St. Louis, MO, USA) and were stained with haematoxylin-eosin for a previous observation.

### Immunohistochemistry

The details, suppliers and concentrations of antibodies used are reported in Table 1. Each antibody was assayed in at least five sections of each ovary from each individual. A streptavidin-biotin immunoperoxidase method was performed as previously described [17,26]. Briefly, after deparaffinization, microwave pre-treatment (antigen retrieval) was performed by incubating the sections in 0.01 M citrate buffer (pH 6.0). The endogen peroxidase activity was inhibited with 1% H_2_O_2 _and nonspecific binding was blocked with 10% (v/v) normal goat serum. All sections were incubated with the primary antibodies for 18 h at 4°C and then for 30 min at room temperature with biotinylated secondary antibodies (preabsorbed with rat serum to avoid unspecific binding) selected specifically for each of the two types of primary antibodies used (mono or polyclonal). Visualization of the antigens was achieved by the streptavidin-peroxidase method (BioGenex, San Ramon, CA) and 3.3-diaminobenzidine (Liquid DAB-Plus Substrate Kit – Zymed, San Francisco, CA) was used as the chromogen. Finally, the slides were washed in distilled water and counterstained with Mayer's haematoxylin, dehydrated and mounted. The different follicular categories studied were classified according to the Nomina Histologica [27]. We analysed tertiary healthy follicles and atretic follicles type I, II or III [4] in both groups. We also assessed cystic follicles in treated rats.

**Table 1:** Used antibodies, suppliers, antigen retrieval and dilutions.

**Antibodies**	**Clone/Source**	**IHC antigen retrieval**	**Dilution**
*Primary antibodies*			
PCNA	PC-10. Novocastra (UK)	Buffer Citrate pH 6. Microwave.	1:100
Ki-67	MIB-5. Dako (Denmark)	Buffer Citrate pH 6. Microwave.	1:60
Active Caspase-3	Polyclonal. R&D systems (USA)	Without antigen retrieval	1:500
Bax	Polyclonal. PU347-UP-Biogenex (San Ramon, CA, USA)	Without antigen retrieval	1:30
Bcl-2	Clone 100. Zymed (San Francisco, CA, USA)	Without antigen retrieval	1:50
Bcl-w	Polyclonal. ab 13525-Abcam (Cambridge, UK)	Without antigen retrieval	1:100
Bcl-xL	Polyclonal. ab 45002-Abcam (Cambridge, UK)	Without antigen retrieval	1:500
*Secondary antibodies*			
Anti-rabbit IgG	Goat Polyclonal. 65-6140-Zymed (San Francisco, CA, USA)	-	1:100
Anti-mouse IgG	Goat Polyclonal. AP181B Chemicon (Temecula, CA, USA)	-	1:100

To verify immunoreaction specificity, adjacent control sections were subjected to the same immunohistochemical method replacing primary antibodies with rabbit and mouse non-immune sera. The specificity of the secondary antibodies was tested by incubation with primary antibodies raised against human antigens with a proven negative reaction with the rat tissues: anti-CD45 (Clon: PD7/26 & 2B11; Dako, Carpinteria, CA) and anti-Ki-67 (polyclonal, rabbit anti-human Ki-67; Dako, Carpinteria, CA). To exclude the possibility of non-suppressed endogenous peroxidase activity some sections were incubated with DAB reagent alone.

### Identification of apoptotic nuclei by TUNEL assay

Apoptotic nuclei were identified using the ApopTag^® ^Plus Peroxidase *In Situ *Apoptosis Detection Kit (Chemicon) according to the manufacturer's protocol. Endogenous peroxidase was blocked by immersing slides in 3% hydrogen peroxide. Negative controls were treated in the same manner except that the TdT labelling enzyme was omitted [28]. Apoptotic nuclei were visualized with diaminobenzidine (DAB) as the chromogen substrate (Biogenex, San Ramon, CA), and counterstained with haematoxylin. Cells showing dark brown staining from the colorimetric reaction were considered positive for DNA fragmentation [29].

### Image analysis

Image analysis was performed using an Image Pro-Plus 3.0.1 system (Media Cybernetics, Silver Spring, MA, USA). For the immunohistochemistry technique, images were digitized by a CCD colour video camera (Sony, Montvale, NJ, USA) mounted on a conventional light microscope (Olympus BH-2, Olympus Co., Japan), using an objective magnification of × 40. The microscope was prepared for Koehler illumination. This was achieved by recording a reference image of an empty field for the correction of unequal illumination (shading correction) and by calibrating the measurement system with a reference slide to determine background threshold values. The reference slides contained a series of tissue sections stained in the absence of a primary antibody. The positive controls were used as inter-assay controls to maximize the levels of accuracy and robustness of the method [18,26,30].

The methodological details of image analysis as a valid method for quantifying expression levels have been described previously [18,26,31-33]. The major strength of the imaging approach used in this study is visualization of the *in situ *localization of proteins within cells of interest. In the past decade, computerized image analysis systems have been developed to obtain objective and accurate quantification of biological markers [26,34].

The image analysis score was calculated separately in each follicular wall layer (granulosa theca interna and theca externa) from at least 50 images of the following structures: tertiary, atretic (type I, II or III), and cystic follicles from ovaries of both groups.

TUNEL, Ki-67 and PCNA staining were evaluated by counting positive cells/total cells for each layer to obtain an index of positive cells. For PCNA marker, only intense positive nucleuses were considered positive.

The immunohistochemical-stained area (IHCSA) for the determination of caspase-3, bax, Bcl-xL, Bcl-w and bcl-2 immunohistochemistry was used. The IHCSA was calculated as a percentage of the total area evaluated through the colour segmentation analysis, which extracts objects by locating all objects of the specific colour (brown stain). The brown stain was selected with a sensitivity of 4 (maximum 5) and a mask was then applied to separate the colours permanently. The images were then transformed to a bi-level scale TIFF format.

### Statistics

The number of individuals per group was obtained from a sample size calculation that evaluated the number of individuals necessary to produce an estimate of the immunoreactivity that would fall within 0.4 units of the real value. The formula used was: n = Z2*SD2/d2, where n = sample size, Z = level of confidence (1.96 for 95%); SD = standard deviation (0.3); d = 0.4. As we were able to reject the null hypothesis in most cases, type 2 errors were not considered a problem.

A statistical software package (SPSS 11.0 for Windows, SPSS Inc., Chicago, IL, USA) was used to perform the statistical tests. The differences between the groups of data were assessed by one-way ANOVA, followed by Duncan's multiple range tests. P < 0.05 values was considered significant. Results were expressed as mean ± SEM.

## Results

The successful induction of the disease was confirmed by observation of ovarian morphology. In treated animals were observed healthy developing follicles, follicles showing different degrees of atresia, many large cysts with thickened granulosa cell layer or with scant granulosa cells; and hypertrophic interstitial glands. Corpora lutea were absent in all cases. Ovaries from control animals exhibited follicles in various stages of development including primary, secondary and tertiary follicles, as well as atretic follicles, interstitial glands and corpora lutea.

### Cell proliferation

**Figure 1:**
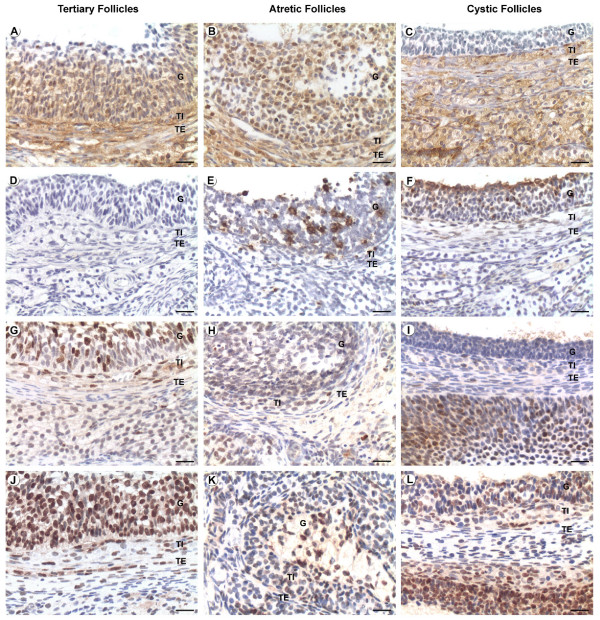
**Localization of caspase-3, Ki-67 and PCNA by immunohistochemistry and in situ apoptosis by TUNEL**. Positive staining is shown as brown colouring of the cytoplasm/nucleus of the cells. Figures A, D, G and J correspond to healthy tertiary follicles; B, E, H and K correspond to atretic follicles and C, F, I and L correspond to cystic follicles. (A-C) caspase-3 immunolocalization, (D-F) TUNEL, (G-I) Ki-67 immunolocalization and (J-L) PCNA immunolocalization. G: Granulosa, TI: Theca Interna, TE: Theca Externa. Bars = 20 μm.

The proliferation index was evaluated by PCNA and Ki-67. Only nuclear staining was found with both cellular proliferation markers while the quantity of PCNA-positive cells was higher than that of Ki-67, possibly due to their higher sensitivity. Both antibodies showed similar patterns in the different follicular categories. Granulosa cells of tertiary follicles from the control group (Figures 1G and 1J) showed a higher proliferation index in relation to tertiary and cystic follicles from the treated group [See Additional file 1]. In theca interna cystic follicles presented lower proliferation index when compared with tertiary follicles in both groups [See Additional file 2] (Figures 1I and 1L). Cystic and atretic follicles showed minor proliferation in theca interna layer (Figures 1H, I, K and 1L). The theca externa also had reduced proliferation index in atretic follicles of both groups and in cystic follicles [See Additional file 3] (Figures 1H, I, K and 1L).

### Cell death

#### TUNEL

The highest percentage of positive cells was found in granulosa cells of atretic follicles type II in both groups [See Additional file 1] (Figure 1E). Tertiary follicles in both groups presented few marked cells (Figure 1D). The cysts exhibited a similar staining to the type I and III atretic follicles (Figure 1F). The theca interna had a few positive cells in all follicular categories, except in tertiary follicles of the treated group, which presented higher staining in relation to tertiary follicles of control animals [See Additional file 2]. Cysts remained at similar levels without significant differences into regard to tertiary follicles in either group (Figure 1D and 1F). Atretic follicles type II of the COD rats presented high levels of DNA fragmentation (Figure 1E). No differences were found in the theca externa between either follicular categories or groups (see additional file 3 for Immunohistochemical analysis of various proliferation and apoptotic proteins and DNA fragmentation in theca externa cells of rats with COD and controls).

#### Active caspase-3

Immunostaining for active caspase-3 was observed in cellular nucleus and cytoplasm. In the granulosa, the most intense staining was in the type II atretic follicles of treated rats, and the least intense was in the tertiary follicles of both groups and cystic follicles (p < 0.05) [See Additional file 1] (Figures 1A–C). In the theca interna, higher expression was observed in the type I atretic follicles in both groups and type II of the light-exposed group [See Additional file 2]. Imunostaining in the theca externa was relatively low with no significant differences between follicular categories or groups [See Additional file 3].

#### Bcl-2 members

**Figure 2:**
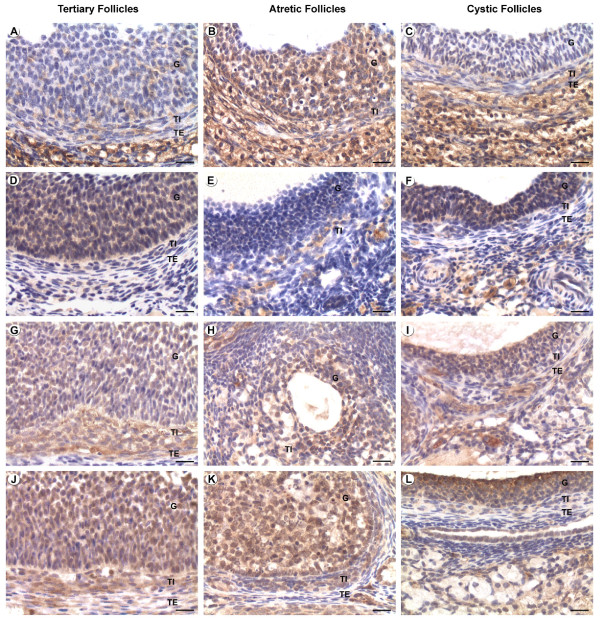
**Immunohistochemical localization of various Bcl-2 family proteins in healthy, atretic and cystic follicles of normal cycling and COD rats**. Positive staining is shown as brown colouring of the cytoplasm of the cells. Figures A, D, G and J correspond to healthy tertiary follicles; B, E, H and K correspond to atretic follicles and C, F, I and L correspond to cystic follicles. (A-C) Bax immunolocalization, (D-F) Bcl-2 immunolocalization, (G-I) Bcl-xL immunolocalization and (J-L) Bcl-w immunolocalization. G: Granulosa, TI: Theca Interna, TE: Theca Externa. Bars = 20 μm.

The granulosa cells presented high expression of antiapoptotic protein in the tertiary and cystic follicles (Figures 2D, F, G, I, J and 2L) with diminishing intensity in the atretic follicles of all categories [See Additional file 1] (Figures 2E and 2H), although intensity of Bcl-w was maintained in the atretic follicles of the control (Figure 2K) and light-exposed group, the highest expression was observed in the cystic and type III atretic follicles of the light-exposed group. In contrast, the expression of the pro-apoptotic protein Bax was weak in the healthy and cystic follicles (Figures 2A and 2C), higher in the type I and II atretic follicles (Figure 2B) and reduced in those of type III where the apoptotic granulosa cells had already been eliminated. In the theca interna Bcl-2 expression was the almost same as the pattern found in the granulosa [See Additional file 2] (Figures 2D to 2F); Bcl-xL expression did not differ between groups (Figures 2G to 2I); the atretic follicles type I of the light-exposed group showed the lowest levels of expression of Bcl-w and those type II showed higher levels of expression with regard to tertiary follilces of the two groups and cystic follicles.

On the other hand, the expression of Bax in this layer was higher in the tertiary follicles of the treated animals than those of cystic and type-I atretic follicles. The values for cystic follicles were similar to those in the tertiary follicles of controls (Figures 2A to 2C). The theca externa showed low expression of the pro and anti-apoptotic proteins with few differences between categories [See Additional file 3].

## Discussion

In the present study we observed significant differences in the apoptosis and proliferation rate in follicles from rats with induced ovarian cysts. Cellular proliferation quantified by means of the immunodetection of PCNA and Ki-67 was higher in the granulosa cells of the healthy follicles in the control group than in the healthy and cystic follicles of light-exposed group. In theca cells, proliferation was lower in cysts than in tertiary follicles. Atretic follicles of all categories presented scarce proliferation indeces with Ki-67. The highest levels in proliferation found in atretic follicles with PCNA in both groups were at the beginning of the process of atresia (stadium I atretic follicles). This is probably because these follicles still express this protein (that has a prolonged half life) although some of their cells are already in elimination through the programmed cell death.

In bovine COD, Isobe and Yoshimura [23,35] found a low index of proliferation in all follicular layers of cysts. They observed intense proliferation in the basal area of the granulosa of normal tertiary follicles, and a decrease in the atretic and cystic follicles. These results are consistent with those found in cystic follicles induced in bovines by ACTH [36] and in rats by oestradiol valerate [37]. However, our results on proliferation conflict with those of Das et al. [38] who found that proliferation indices in the cystic follicles of women with PCOD were high, which was attributed by the authors to the high levels of androgens that occur in the human disease. It should be taken into account that androgens are at normal levels in cows with COD [39] and that in the model used in this work, testosterone was even at levels below that found in rats in proestrus (unpublished data). It is known that the granulosa cells proliferate before the cyclic recruitment of the follicles under the influence of the gonadotropins, achieving high rates of mitosis in the total absence of these hormones. However, the final stage of development prior to ovulation is exclusively dependent on gonadotropins [40,41]. There are reports that indicate that cellular proliferation in the granulosa is regulated by FSH, oestrogens and insulin as well as some growth factors [41-43]. Androgens produced by the ovarian theca-interstitial cells play a decisive regulatory function in folliculogenesis because they serve as the precursors of estrogen synthesis in granulosa cells. In contrast, oestrogens enhance the responsiveness of ovarian follicles to gonadotropin stimulation and increase granulosa cell proliferation. However, excess androgen production impairs follicular function: it inhibits the effect of oestrogen on follicular growth, inhibits FSH induction of LH receptors in granulosa cells [44] and increases atresia among the follicles in rat ovaries [45,46]. We have demonstrated that DNA fragmentation, as well as activated caspase-3, and Bax protein expression were significantly lower in all layers of tertiary and cystic follicles from COD rats than in normal atretic follicles from both groups, while the expression of survival proteins of the Bcl-2 family such as Bcl-2, Bcl-xL and Bcl-w was high in healthy and cystic follicles in both groups. Although, the cystic follicles showed moderate levels in the DNA fragmentation, these levels were not as high as those that showed the follicles with advanced degree of atresia. This is probably due to that certain percentage of cells of the cystic follicles degenerates by apoptosis without affects the structure of these follicles that stay through the time conserving the antrum, contrary to that observed in the process of follicular atresia, in which the follicles go reducing its size and the antrum disappears early. Anderson and Lee [47] found that during cystogenesis in a dehydroepiandrosterone (DHEA) induction model, apoptosis systematically progresses from the cumulus towards the mural granulosa layer and the outer layer of mural granulosa cells escapes apoptosis. In contrast, granulosa cells of atretic follicles undergo apoptosis in a random manner. The relating results to the high expression of Bcl-w in the cystic follicles could indicate the tendency of these to remain through the time. However there is maintenance in the expression of this protein in the healthy and atretic follicles what would be indicating Bcl-w doesn't have an important role in the survival of ovarian cells, contrarily to Bcl-2 and the Bcl-xL. On the other hand it is necessary to consider that exists a balance among pro and anti-apoptotic components that it can be altered and consequently an overexpression of pro-apoptotic elements (such as bax) it would originate a desbalance that would take to the cells in this condition toward the apoptosis. Isobe and Yoshimura [23,35,48] found low proliferation and apoptosis in cystic follicles in cows when compared with healthy follicles. These data are consistent with those of other authors who, using the TUNEL method and immunohistochemistry for the detection of caspase-3 and Bax in an experimental model of COD in bovines also found a lower index of proliferation and apoptosis in ovarian cysts than in healthy follicles [36]. In studies carried out in women with PCOD, Das et al [38] found high mRNA and protein expression of antiapoptotic factors such as cIAP-2 and Bcl-xL and lower expression of Bax and Caspase-3 in granulosa cells of cystic follicles, with similar values to those found in healthy follicles. On the other hand, Almahbobi et al. [49] found that granulosa cells from ovarian cysts of women with COD are normal, with low levels of apoptosis and high expression of gonadotropin receptors, specifically FSHr.

The response of the ovarian follicle to the combined effect of survival and death factors determines its ultimate fate: follicular atresia or ovulation. Progesterone is one of the factors that is induced by LH and that was reported to act as an antiapoptotic factor in luteinized rat and human granulosa cells [50]. Gonadotropins were also demonstrated to affect the apoptotic machinery by suppressing the expression of proapoptotic proteins [9,51] as well as inducing the expression of antiapoptotic proteins [50]. However, Yacobi et al [50,52] found that although gonadotropins (principally LH) decrease apoptosis in granulosa cells in cultured rat preovulatory follicles they increase apoptosis in theca/interstitial cells through the caspase-3 cascade. Tilly et al. [9] demonstrated that the inhibition of granulosa cell apoptosis and follicular atresia mediated by gonadotropin treatment may be linked to the ability of gonadotropins to reduce the amount of Bax present in granulosa cells, while maintaining a constitutive level of Bcl-2 and Bcl-xL expression. Moreover, the level of Bcl-xS mRNA is reduced by gonadotropin treatment and this effect may contribute to the shift in the balance of death inducer to death repressor gene expression. It is also possible that other hormonal signalling such as ovarian steroids or locally produced growth factors that can influence granulosa cell fate [9,53,54], serve as the primary regulators of Bcl-2 and Bcl-xL gene expression. It is interesting that Bax-deficient mice have abnormal follicles with an excessive number of granulosa cells [55]. Although there were no changes in the levels of serum gonadotropins in the treated animals [32], their constant levels (without the recurrent variations characteristic of the oestrus cycle) probably affect the expression of pro and anti-apoptotic proteins of the Bcl-2 family leading to follicular persistence over time.

We found moderate immunostaining of caspase-3 in the theca cell layer in all follicular categories in both groups. The role of caspase-3 in rat ovarian theca cells is not clear, since this cell type does not typically undergo apoptosis [4,9,56,57]. It is possible that caspase-3 is activated in theca cells but does not lead to apoptosis because these cells lack the endogenous DNase I necessary to complete the apoptotic programme [56].

All the data presented here indicate that the cells of the follicular cysts are functional and, in fact, several authors have demonstrated this functionality, through cellular production of hormones, enzymes and their response to different stimuli [49,58,59].

## Conclusion

Although much remains to be done in order to characterize the pathogenesis of cystic ovaries, we have confirmed that cellular proliferation and apoptosis are altered in cystic follicles of rats, as occurs in related diseases in different species. These results support the concept that the combination of weak proliferative activity and low levels of apoptosis observed in the follicular wall of cyst, could explain why the cystic follicles grow slowly and then maintain a static condition without degeneration, which leads to their persistence. These alterations may be due to structural and functional modifications that take place in these cells and could be related to the hormonal changes that happen in animals with this disease. Further studies will be needed to assess the specific role and regulation of each one of these cellular components and their participation in cystogenesis.

## Competing interests

The authors declare that they have no competing interests.

## Authors' contributions

NRS carried out the immunohistochemistry and TUNEL studies, participated in the data analysis and drafted the manuscript. CGP and NSA participated in the design of the study and helped to collect and process the samples. EJG participated in the design of the study and helped to draft the manuscript. LGN participated in the selection of animals, experimental model and collection of the samples. HHO conceived of the study, and participated in its design and coordination, participated in the data analysis and helped to draft the manuscript. All authors read and approved the final manuscript.

## Supplementary Material

Additional file 1 **Table S1**. Immunohistochemical analysis of various proliferation and apoptotic proteins and DNA fragmentation in granulosa cells of rats with COD and controls. Click here for file

Additional file 2 **Table S2**. Immunohistochemical analysis of various proliferation and apoptotic proteins and DNA fragmentation in theca interna cells of rats with COD and controls. Click here for file

Additional file 3 **Table S3**. Immunohistochemical analysis of various proliferation and apoptotic proteins and DNA fragmentation in theca externa cells of rats with COD and controls. Click here for file
