# Supplementary material for: An imbalance between apoptosis and proliferation contributes to follicular persistence in polycystic ovaries in rats
Source: Reprod Biol Endocrinol. 2009 Jul 1;7:68. doi: 10.1186/1477-7827-7-68 (PMC2713246; doi:10.1186/1477-7827-7-68)
Supplement: Additional file 1 — Table S1. Immunohistochemical analysis of various proliferation and apoptotic proteins and DNA fragmentation in granulosa cells of rats with COD and controls. [file 1477-7827-7-68-S1.doc]

**Table S1.** Immunohistochemical analysis of various proliferation and apoptotic proteins and DNA fragmentation in granulosa cells of rats with COD and controls.

|  | **Proliferation** | |  | **Apoptosis** | | | | | | |
| --- | --- | --- | --- | --- | --- | --- | --- | --- | --- | --- |
|  | **PCNA*** | **Ki-67*** |  | **TUNEL*** | **Caspase-3**** |  | | | | |
|  |  |  |  |  |  | **Bcl-2 family Anti-apoptotic** | | |  | **Pro-apoptotic** |
|  |  |  |  |  |  | **Bcl-2**** | **Bcl-xL**** | **Bcl-w**** |  | **Bax**** |
| **Control group** |  |  |  |  |  |  |  |  |  |  |
| Tertiary follicles | 16.12+/-1.06^a^ | 23.96+/-2.19^a^ |  | 0.46+/-0.16^a^ | 2.76+/-1.01^a^ | 18.08+/-2.43^a^ | 23.56+/-3.16^ab^ | 33.82+/-6.76^ab^ |  | 1.41+/-0.12^a^ |
| Atretic follicles type I | 7.02+/-0.09^bc^ | 4.69+/-1.49^c^ |  | 5.06+/-0.75^bc^ | 11.40+/-1.40^bc^ | 4.29+/-0.69^b^ | 35.49+/-2.88^b^ | 28.83+/-4.17^b^ |  | 8.44+/-3.71^b^ |
| Atretic follicles type II | 5.13+/-0.71^cd^ | 2.31+/-1.40^c^ |  | 13.16+/-0.92^d^ | 15.19+/-1.61^c^ | 4.37+/-0.90^b^ | 23.31+/-3.32^ab^ | 29.31+/-6.97^b^ |  | 7.20+/-3.01^b^ |
| Atretic follicles type III | 3.02+/-0.61^d^ | 0.81+/-0.39^c^ |  | 9.13+/-1.69^cd^ | 12.45+/-1.99^c^ | 7.08+/-3.10^b^ | 15.44+/-4.06^a^ | 35.60+/-3.30^ab^ |  | 5.04+/-0.91^ab^ |
|  |  |  |  |  |  |  |  |  |  |  |
| **Light exposed group** |  |  |  |  |  |  |  |  |  |  |
| Tertiary follicles | 6.72+/-0.99^bc^ | 13.19+/-1.92^b^ |  | 2.89+/-0.62^ab^ | 4.69+/-1.06^ab^ | 17.02+/-2.36^a^ | 26.83+/-2.74^ab^ | 37.82+/-2.17^ab^ |  | 1.94+/-0.59 ^a^ |
| Cystic follicles | 2.41+/-0.61^d^ | 1.11+/-0.52^c^ |  | 9.33+/-0.94^cd^ | 3.18+/-0.42^a^ | 16.09+/-0.94^a^ | 26.66+/-2.52^ab^ | 43.779+/-2.35 ^a^ |  | 2.09+/-0.34 ^a^ |
| Atretic follicles type I | 9.05+/-1.93^b^ | 4.54+/-1.38^c^ |  | 5.06+/-0.75^bc^ | 14.16+/-4.16^c^ | 2.79+/-0.15^b^ | 25.20+/-5.90^ab^ | 31.07+/-1.177^b^ |  | 7.31+/-0.01^b^ |
| Atretic follicles type II | 3.01+/-1.21^d^ | 2.57+/-1.27^c^ |  | 20.15+/-4.15^e^ | 24.10+/-8.74^d^ | 4.19+/-0.90^b^ | 21.09+/-4.37^a^ | 35.39+/-0.33^ab^ |  | 7.47+/-1.74^b^ |
| Atretic follicles type III | 3.57+/-0.78^d^ | 1.51+/-0.58^c^ |  | 7.09+/-1.27^bc^ | 8.77+/-2.48^abc^ | 3.75+/-0.78^b^ | 16.25+/-2.50^a^ | 43.082+/-1.59^a^ |  | 5.65+/-2.08^ab^ |
| The values represent Mean +/- Standard Error of Mean. *percentage of positive cells. **IHCSA: immunohistochemical stained area. ^a-e^ Values in the same column with different superscripts differ. (p<0.05). | | | | | | | | | | |
