# Supplementary material for: An imbalance between apoptosis and proliferation contributes to follicular persistence in polycystic ovaries in rats
Source: Reprod Biol Endocrinol. 2009 Jul 1;7:68. doi: 10.1186/1477-7827-7-68 (PMC2713246; doi:10.1186/1477-7827-7-68)
Supplement: Additional file 2 — Table S2. Immunohistochemical analysis of various proliferation and apoptotic proteins and DNA fragmentation in theca interna cells of rats with COD and controls. [file 1477-7827-7-68-S2.doc]

**Table S2.** Immunohistochemical analysis of various proliferation and apoptotic proteins and DNA fragmentation in theca interna cells of rats with COD and controls.

|  | **Proliferation** | |  | **Apoptosis** | | | | | | |
| --- | --- | --- | --- | --- | --- | --- | --- | --- | --- | --- |
|  | **PCNA*** | **Ki-67*** |  | **TUNEL*** | **Caspase-3**** |  | | | | |
|  |  |  |  |  |  | **Bcl-2 family Anti-apoptotic** | | |  | **Pro-apoptotic** |
|  |  |  |  |  |  | **Bcl-2**** | **Bcl-xL**** | **Bcl-w**** |  | **Bax**** |
| **Control group** |  |  |  |  |  |  |  |  |  |  |
| Tertiary follicles | 12.67+/-1.67^a^ | 10.62+/-1.41^a^ |  | 0.22+/-0.22^a^ | 8.84+/-2.60^a^ | 6.98+/-0.83^ab^ | 13.04+/-3.13 | 20.02+/-5.13^acd^ |  | 1.81+/-0.27^a^ |
| Atretic follicles type I | 9.03+/-2.00^bc^ | 7.06+/-3.41^ab^ |  | 0.50+/-0.25^a^ | 18.96+/-1.96^b^ | 1.89+/-1.29^c^ | 12.53+/-1.11 | 22.08+/-3.52^ade^ |  | 4.45+/-1.72^ab^ |
| Atretic follicles type II | 5.13+/-0.47^de^ | 3.52+/-1.18^b^ |  | 1.83+/-0.82^ab^ | 8.02+/-0.97^a^ | 1.66+/-0.34^c^ | 8.41+/-2.91 | 14.62+/-4.71^abc^ |  | 4.26+/-1.07^ab^ |
| Atretic follicles type III | 2.02+/-0.63^e^ | 1.25+/-0.27^b^ |  | 1.86+/-0.38^ab^ | 8.92+/-1.93^a^ | 15.49+/-3.29^d^ | 8.16+/-1.89 | 25.62+/-3.72^de^ |  | 7.17+/-4.01^bc^ |
|  |  |  |  |  |  |  |  |  |  |  |
| **Light exposed group** |  |  |  |  |  |  |  |  |  |  |
| Tertiary follicles | 11.72+/-1.13^ab^ | 9.69+/-2.37^a^ |  | 3.41+/-1.19^b^ | 6.72+/-2.09^a^ | 5.51+/-1.02^abc^ | 8.97+/-2.00 | 15.50+/-2.24^abc^ |  | 10.66+/-1.53^cd^ |
| Cystic follicles | 3.92+/-0. 89 ^e^ | 4.57+/-1.89^b^ |  | 1.97+/-0.14^ab^ | 6.22+/-1.40^a^ | 7.74+/-0.76^a^ | 9.31+/-2.10 | 11.15+/-1.85^bc^ |  | 5.21+/-1.04^ab^ |
| Atretic follicles type I | 7.80+/-1. 91 ^dc^ | 3.42+/-1.14^b^ |  | 0.50+/-0.25^a^ | 16.47+/-1.53^b^ | 1.51+/-0.05^c^ | 10.95+/-5.92 | 9.83+/-1.49^b^ |  | 3.24+/-0.00^ab^ |
| Atretic follicles type II | 2.79+/-0.50 ^e^ | 5.57+/-1.16^b^ |  | 3.82+/-1.48^b^ | 16.63+/-3.48^b^ | 3.24+/-1.94^bc^ | 6.34+/-0.63 | 31.30+/-1.89^e^ |  | 7.30+/-1.32^bc^ |
| Atretic follicles type III | 2.72+/-1.20 ^e^ | 5.98+/-1.18^b^ |  | 1.62+/-0.76^ab^ | 6.60+/-3.30^a^ | 1.77+/-0.19^c^ | 6.01+/-1.13 | 19.14+/-1.43^abcd^ |  | 14.41+/-1.62^d^ |
| The values represent Mean +/- Standard Error of Mean. *percentage of positive cells. **IHCSA: immunohistochemical stained area. ^a-e^ Values in the same column with different superscripts differ. (p<0.05). | | | | | | | | | | |
