# Supplementary material for: An imbalance between apoptosis and proliferation contributes to follicular persistence in polycystic ovaries in rats
Source: Reprod Biol Endocrinol. 2009 Jul 1;7:68. doi: 10.1186/1477-7827-7-68 (PMC2713246; doi:10.1186/1477-7827-7-68)
Supplement: Additional file 3 — Table S3. Immunohistochemical analysis of various proliferation and apoptotic proteins and DNA fragmentation in theca externa cells of rats with COD and controls. [file 1477-7827-7-68-S3.doc]

**Table S3.** Immunohistochemical analysis of various proliferation and apoptotic proteins and DNA fragmentation in theca externa cells of rats with COD and controls.

|  | **Proliferation** | |  | **Apoptosis** | | | | | | |
| --- | --- | --- | --- | --- | --- | --- | --- | --- | --- | --- |
|  | **PCNA*** | **Ki-67*** |  | **TUNEL*** | **Caspase-3**** |  | | | | |
|  |  |  |  |  |  | **Bcl-2 Family Anti-apoptotic** | | |  | **Pro-apoptotic** |
|  |  |  |  |  |  | **Bcl-2**** | **Bcl-xL**** | **Bcl-w**** |  | **Bax**** |
| **Control group** |  |  |  |  |  |  |  |  |  |  |
| Tertiary follicles | 3.51+/-0.64^a^ | 1.81+/-0.28^abc^ |  | 0.12+/-0.12 | 1.38+/-0.56 | 2.00+/-0.40^a^ | 1.18+/-0.41^a^ | 2.87+/-0.62^a^ |  | 0.31+/-0.11^a^ |
| Atretic follicles type I | 0.93+/-0.54^b^ | 1.42+/-0.12^abc^ |  | 0.27+/-0.27 | 0.00+/-0.00 | 0.33+/-0.20^a^ | 1.56+/-0.57^a^ | 5.08+/-1.70^ab^ |  | 0.63+/-0.43^a^ |
| Atretic follicles type II | 0.87+/-0.87^b^ | 1.06+/-0.49^abc^ |  | 0.54+/-0.19 | 2.08+/-2.08 | 0.99+/-0.49^a^ | 1.26+/-0.60^a^ | 1.18+/-0.56^a^ |  | 1.99+/-1.71^ab^ |
| Atretic follicles type III | 0.80+/-0.50^b^ | 0.34+/-0.34^a^ |  | 0.48+/-0.26 | 2.65+/-1.05 | 4.70+/-2.22^b^ | 2.29+/-0.90^a^ | 3.66+/-1.19^ab^ |  | 1.74+/-1.17^ab^ |
|  |  |  |  |  |  |  |  |  |  |  |
| **Light exposed group** |  |  |  |  |  |  |  |  |  |  |
| Tertiary follicles | 4.64+/-0.90^a^ | 2.48+/-0.58^c^ |  | 0.28+/-0.28 | 2.33+/-0.83 | 2.49+/-0.70^ab^ | 1.29+/-0.70^a^ | 2.28+/-0.29^a^ |  | 2.90+/-0.19^ab^ |
| Cystic follicles | 0.82+/-0.39^b^ | 0.89+/-0.35^ab^ |  | 0.28+/-0.18 | 1.62+/-0.73 | 2.32+/-0.57^ab^ | 1.46+/-0.51^a^ | 1.45+/-0.29^a^ |  | 1.97+/-0.64^ab^ |
| Atretic follicles type I | 0.48+/-0.48^b^ | 0.47+/-0.29^a^ |  | 0.27+/-0.27 | 0.00+/-0.00 | 0.70+/-0.06^a^ | 5.00+/-0.01^b^ | 2.19+/-0.57^a^ |  | 1.73+/-0.00^ab^ |
| Atretic follicles type II | 0.83+/-0.83^b^ | 2.19+/-0.59^bc^ |  | 0.01+/-0.01 | 3.97+/-0.46 | 2.00+/-1.62^a^ | 0.42+/-0.19^a^ | 6.96+/-2.33^b^ |  | 4.23+/-1.38^b^ |
| Atretic follicles type III | 1.00+/-1.00^b^ | 0.66+/-0.35^ab^ |  | 0.12+/-0.12 | 6.20+/-6.20 | 1.12+/-0.15^a^ | 0.78+/-0.50^a^ | 4.46+/-1.23^ab^ |  | 1.07+/-0.48^ab^ |
| The values represent Mean +/- Standard Error of Mean. *percentage of positive cells. **IHCSA: immunohistochemical stained area. ^a-c^ Values in the same column with different superscripts differ. (p<0.05). | | | | | | | | | | |
